# Are Wild Boars Exposed to Triclosan and Triclocarban?

**DOI:** 10.3390/ani16182863

**Published:** 2026-09-11

**Authors:** Slawomir Gonkowski, Manolis Tzatzarakis, Elena Vakonaki, Liliana Rytel

**Affiliations:** 1 Department of Clinical Physiology, Faculty of Veterinary Medicine, University of Warmia and Mazury in Olsztyn, Oczapowskiego 13, 10-719 Olsztyn, Poland; 2 Laboratory of Toxicology, School of Medicine, University of Crete, 70013 Heraklion, Crete, Greeceevakonaki@uoc.gr (E.V.); 3 Department and Clinic of Internal Diseases, Faculty of Veterinary Medicine, University of Warmia and Mazury in Olsztyn, 10-719 Olsztyn, Poland; liliana_rytel@wp.pl

**Keywords:** wildlife, endocrine disruptors, environmental pollution, exposure, hair

## Abstract

Triclosan (TCS) and triclocarban (TCC) are antimicrobial compounds widely used in numerous industries, making them significant environmental pollutants that infiltrate living organisms and exhibit endocrine-disrupting activity. However, knowledge regarding the exposure of wild terrestrial mammals to these substances remains virtually non-existent. Therefore, this study analyzed, for the first time, TCS and TCC levels in the hair of wild boars using liquid chromatography-tandem mass spectrometry (LC-MS). Despite widespread environmental contamination with these compounds, concentrations above the limit of detection (LOD: 2.3 pg/mg for TCS and 0.8 pg/mg for TCC) were detected in only a small percentage of the analyzed samples: 14.81% for TCC and 3.70% for TCS. Higher concentrations and a greater frequency of TCC occurrence were observed in animals from highly urbanized and industrialized regions. In contrast, TCC and TCS concentrations in animals from other areas only sporadically exceeded the LOD. These results suggest that wild boars are primarily exposed to TCC in highly urbanized areas. In less-urbanized regions, animal exposure to both compounds is usually below the LOD and likely depends on poorly defined local factors. Nonetheless, fully clarifying the exposure of wild terrestrial mammals to TCC and TCS requires further comprehensive research.

## Simple Summary

Triclosan (TCS) and triclocarban (TCC) are antimicrobial compounds widely used in various industries, primarily in cosmetics manufacturing. Their widespread application leads to significant environmental pollution, and upon entering living organisms, they impair the function of numerous internal organs and biological systems. However, knowledge regarding the exposure of wild terrestrial mammals to these substances remains virtually non-existent. Therefore, this study aimed to assess the exposure of wild boars to TCS and TCC by analyzing their concentrations in hair samples. The results demonstrate that despite the ubiquitous presence of TCC and TCS in the environment, only a relatively small percentage of the tested samples contained detectable levels of these compounds. A notable exception was observed in animals from highly urbanized areas, where TCC levels were significantly higher than in individuals from other regions. Consequently, this study indicates that wild boars can be exposed to TCC, and to a lesser extent to TCS, although the levels of these substances in hair are usually below the limit of detection.

## 1. Introduction

**Figure 1:**
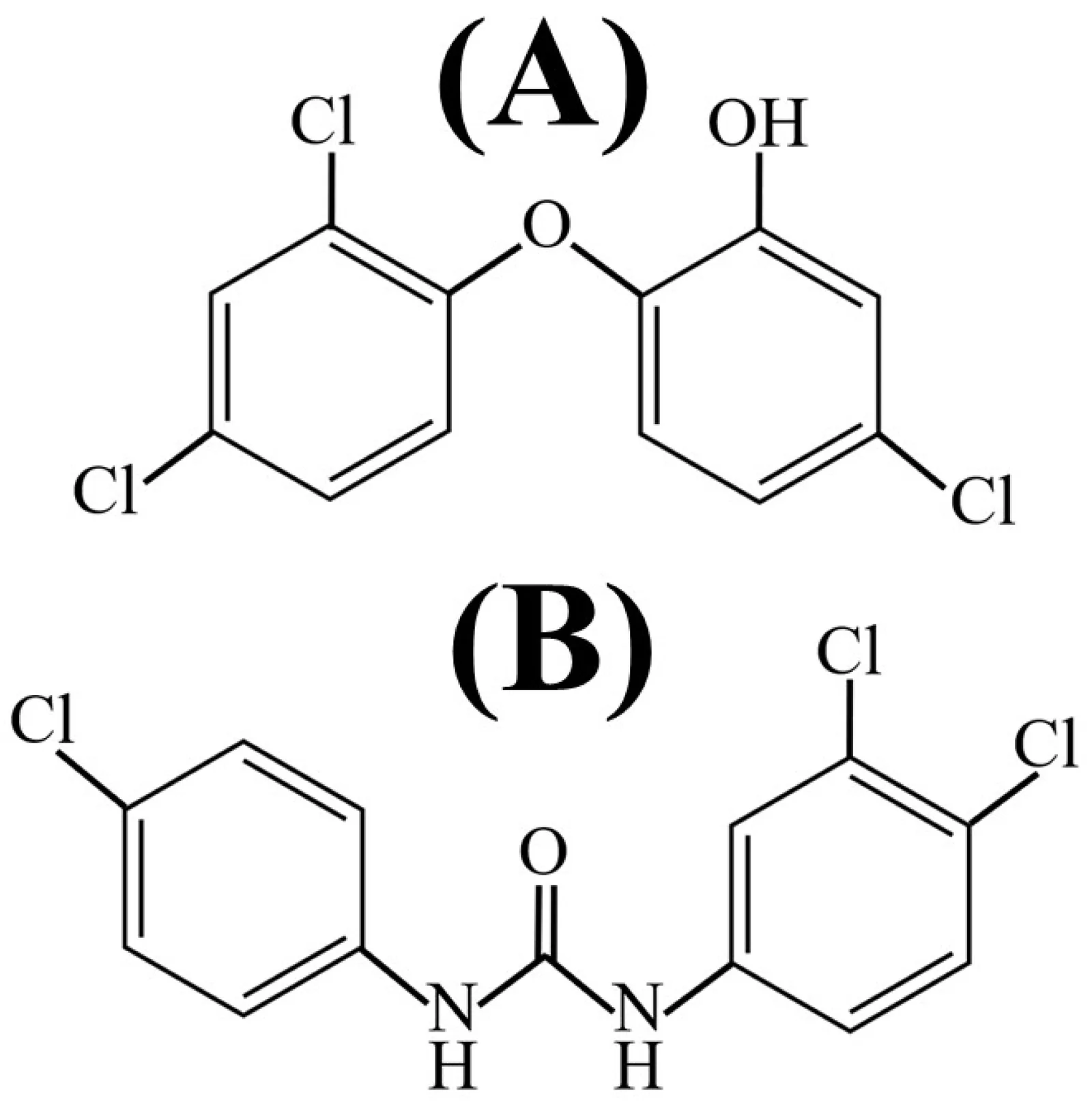
Chemical structure of triclosan (**A**) and triclocarban (**B**).

Triclosan (TCS) and triclocarban (TCC) are aromatic organic compounds characterized by strong antimicrobial activity [1] (Figure 1). Due to these properties, they have been widely utilized in various industries since the 1960s. These compounds are primarily employed in the cosmetics industry as antibacterial and antifungal additives in toothpastes, personal care products, deodorants, shampoos, and shower gels, as well as in liquid soaps (for TCS) and bar soaps (for TCC) [2]. Furthermore, these substances are used in the textile and plastics industries. Incorporating them into sportswear, utensils, toys, and household appliances aims to preserve the products and prevent microbial growth on their surfaces [3,4]. Triclosan and triclocarban are also found in dishwashing liquids and disinfectant solutions used, for instance, for hand sanitization or medical equipment disinfection [4,5].

Although TCS and TCC are characterized by low acute toxicity and were therefore considered neutral to human and animal health for many years, their endocrine-disrupting effects have recently been confirmed [1]. As endocrine-disrupting chemicals, TCS and TCC dysregulate the endocrine system (mainly the production of sex and thyroid hormones), and consequently, disrupt the functioning of many internal organs and systems [1,6]. Above all, these substances negatively affect the male and female reproductive systems, causing reproductive cycle disorders and pregnancy complications in females, as well as reduced sperm viability in males [7,8]. Furthermore, TCS and TCC adversely affect the functioning of the nervous, immune, digestive, and cardiovascular systems [1,6,9,10]. Some studies have confirmed that exposure to TCS and/or TCC increases the risk of cognitive impairment, allergic and inflammatory conditions, and asthma [1,11,12]. Exposure to these substances may also be associated with an increased risk of obesity, diabetes, hypertension, and even neurodegenerative changes [6,13,14,15,16].

Due to such multidirectional harmful effects, some countries have banned the use of TCS and TCC in food contact materials or products intended for children, and have significantly restricted their use in the cosmetics industry [17]. However, despite these restrictions, both TCS and TCC widely contaminate the natural environment worldwide. To date, the presence of TCS and TCC has been confirmed in various environmental matrices, such as surface water, groundwater, soil, and the atmosphere [18,19,20]. The presence of these substances has also been detected in crops and dust [21,22]. Naturally, there are significant regional differences in the degree of environmental contamination with TCS and TCC, which primarily result from the level of industrialization and urbanization, but also from the habits of the population in a given area (e.g., the frequency of cosmetics use) [23,24,25,26]. However, the global scale of the problem associated with the occurrence of TCS and TCC in the environment is evidenced by the fact that these substances have also been detected in areas far removed from densely populated regions, such as Antarctica [27].

The significant degree of environmental contamination with TCS and TCC exposes both humans and animals to these substances, which enter the body through the skin, the gastrointestinal tract, and the respiratory system, as well as through the placenta during the prenatal period [6,28,29]. The vast majority of studies to date have focused on human exposure to these compounds. The presence of both TCS and TCC has been confirmed in various biological matrices. In addition to conventional matrices, such as serum [30] and urine [25,30,31], the presence of these compounds has also been confirmed in human saliva [32], semen [33], amniotic fluid [30,34], hair, and nails [31]. Knowledge regarding the presence of TCS and, to a much lesser extent, TCC, in aquatic organisms is also relatively extensive. To date, TCS has been detected in various tissues of both marine and freshwater fish, marine mammals, and reptiles, as well as in different types of invertebrates [35,36,37,38]. The exposure of aquatic and/or fish-eating birds to triclosan has also been documented [39]. In turn, TCC has been detected in fish tissues [40]. These data confirm the widespread exposure of living organisms to TCS and TCC. Chronic exposure to these compounds can not only directly cause adverse health effects in humans and animals, but can also promote antibiotic resistance among environmental microorganisms [6]. On the other hand, many wild animal species increasingly live near human settlements. One such species is the wild boar, which can often be found even in green spaces located within the boundaries of large cities, foraging in dustbins and landfills [41,42]. The fact that they live in close proximity to human settlements means they may be significantly exposed to anthropogenic environmental pollution [43]. Consequently, the presence of wild boars within city boundaries makes this species a potentially excellent indicator for assessing environmental pollution [43,44]. On the other hand, however, the potential antimicrobial resistance of microorganisms within the animals’ bodies, developed under the influence of chronic exposure to TCS and TCC, may pose a threat to human and domestic animal health [45,46]. Therefore, assessing the exposure of wild animals living near human settlements to TCS and TCC is crucial from the perspective of environmental toxicology.

The choice of hair samples as a matrix for the present study is also not accidental. Environmental pollutants, including TCS and TCC, penetrate the inside of the hair and accumulate there [31,47]. Consequently, the analysis of hair samples allows for the assessment of long-term environmental exposure, in contrast to classic matrices such as blood serum or urine, in which the levels of the substances in question undergo short-term changes [48]. Furthermore, hair samples are easy to collect; they can be sampled after the death of the individual because hair does not undergo autolysis, and they are also easy to store and transport [48]. For this reason, hair samples are gaining increasing importance in assessing the exposure of living organisms to environmental pollutants, including TCS and TCC [31,47,49].

Taking the above facts into consideration, the aim of the present study was to assess the concentrations of TCS and TCC in hair samples of wild boars living in different regions of Poland and thereby to expand the knowledge on the level of environmental pollution by these compounds and the degree of exposure of wild terrestrial mammals to these substances.

## 2. Materials and Methods

### 2.1. Reagents

The following reagents were used in this study: TCS, TCC and ammonium acetate (≥98%) were purchased from Sigma-Aldrich (St. Louis, MO, USA), methanol and acetonitrile (LC-MS grade) from Fisher Chemical (Loughborough, UK) and phenobarbital as internal standard (IS) from Lipomed AG, Arlesheim, Switzerland. Ultrapure water was produced by Merck’s Direct-Q 3UVwater purification system (Darmstadt, Germany).

### 2.2. Sampling

**Figure 2:**
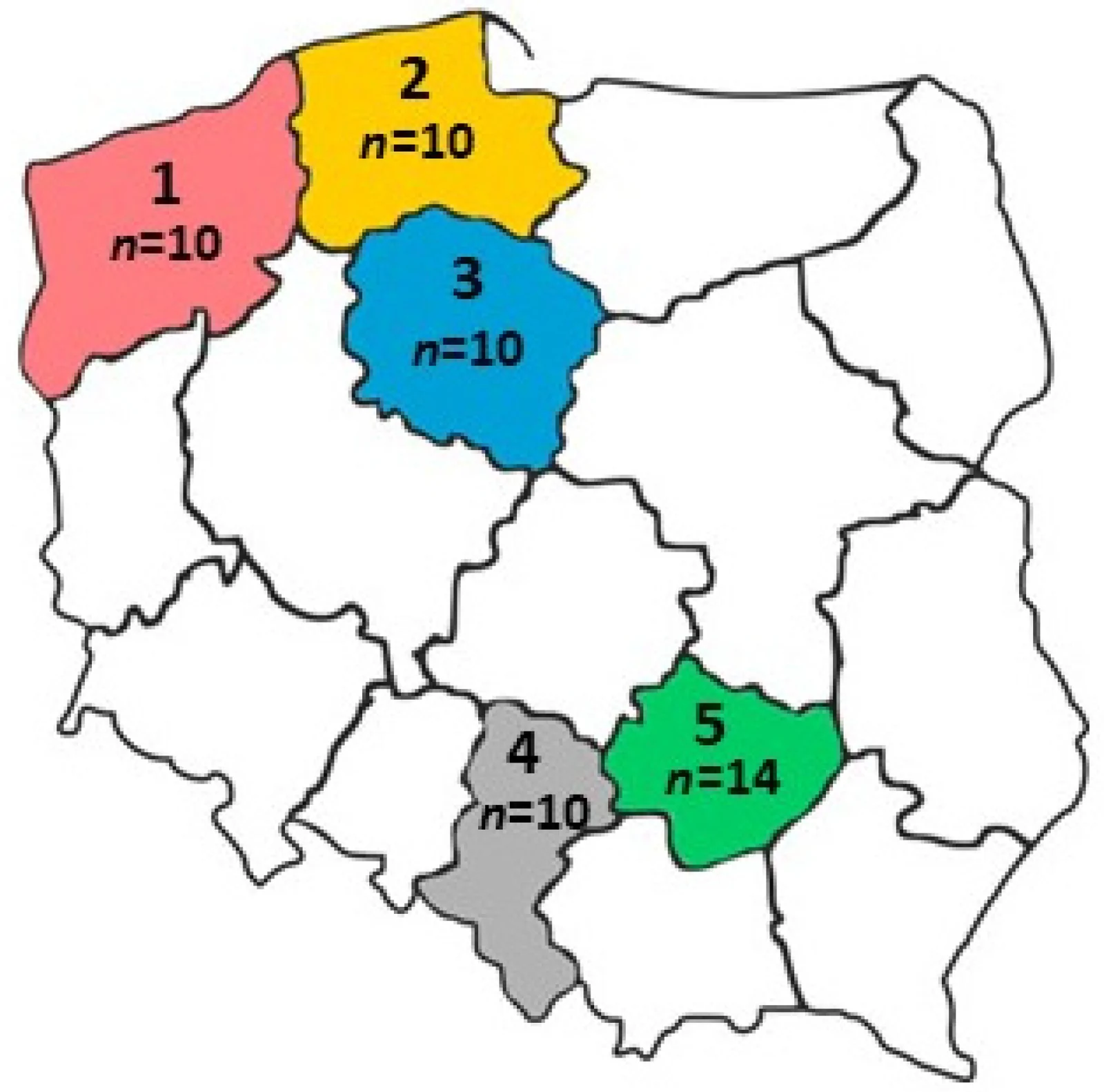
Regions included in the study: 1 West Pomeranian; 2 Pomeranian; 3 Kuyavian–Pomeranian; 4 Silesian; 5 Holy Cross voivodeships, *n* number of samples from each region.

During the study, hair samples collected from 54 adult wild boars of both sexes (29 males and 25 females) were analyzed. The animals were hunted during legal hunts organized in the autumn of 2021 and autumn of 2022 by the Polish Hunting Association and originated from five different regions of Poland, namely: the Pomeranian, Kuyavian–Pomeranian, West Pomeranian, Silesian, and Holy Cross voivodeships (Figure 2). The characteristics of the regions are presented in Table 1.

Hair samples from the undercoat weighing approximately 2 g were collected from the same abdominal area of each animal included in the study, within a maximum of 30 min post-mortem. Immediately after collection, the hair samples were mechanically cleaned of any external debris and wrapped in aluminum foil. Subsequently, the samples were stored at room temperature in the dark until further analysis.

The animals were harvested during legal hunts conducted by licensed hunters in accordance with the Polish Hunting Law and the applicable authorisations issued by the relevant hunting-district lessees or managers. Hair samples were collected post-mortem with the permission of the persons or entities legally entitled to dispose of the harvested animals. No procedures were performed on live animals for the purposes of this study; therefore, approval by a Local Ethics Committee for Animal Experimentation was not required under the Polish Act of 15 January 2015 on the Protection of Animals Used for Scientific or Educational Purposes.

### 2.3. TCS and TCC Extraction

Hair samples were prepared and analyzed according to the method previously described [49,51]. Initially, the samples were washed twice in ultrapure water and twice in methanol in order to eliminate exogenous contamination from the surface of the hair. Following washing, the hair samples were dried at 50 °C and sectioned into fragments of a few millimeters.

For this purpose, 100 mg of each hair sample was placed into glass screw-cap tubes along with 2 × 2 mL of methanol and 25 ng of IS. To avoid contamination, only glass tubes washed with LC–MS—grade methanol and dried at 80 °C were used. Compounds were extracted in an ultrasonic water bath for 2 × 2 h with periodic mixing using a vortex system. The extracts were dried completely under a nitrogen stream sustained at 35 °C. Following the addition of 100 μL of methanol to dissolve the residues, the mixture was transferred to 2 mL vials with inserts. Final analysis was performed using liquid chromatography–mass spectrometry (LC-MS) with an injection volume of 10 μL.

### 2.4. Instrumentation

The detection and quantification of TCS and TCC were performed using an LC–MS system (2010 EV, Shimadzu, Kyoto, Japan) after compound separation on a Supelco Discovery C18 column (250 mm × 4.6 mm, 5 μm; Sigma-Aldrich, St. Louis, MO, USA) at 30 °C.

Chromatographic separation was performed at a flow rate of 0.6 mL/min using 5 mM ammonium acetate (solvent A) and acetonitrile containing 0.1% formic acid (solvent B). To monitor the analytes, an atmospheric pressure chemical ionization (APCI) source and a quadrupole mass filter in negative selected ion monitoring (SIM) mode were used. Retention times were 23.18 min for TCS, 23.0 min for TCC and 14.08 min for IS. In turn, selected *m*/*z* ions were 286.9 and 332.9 for TCS, 360.9 and 314.9 for TCC and 231.1 for IS. The interface, CDL, and heat block temperatures were set at 400 °C, 200 °C, and 200 °C, respectively. The detector voltage was set at 1.5 kV, the drying gas pressure was set at 0.02 MPa and the nebulizing gas flow was set at 2.5 L/min.

### 2.5. Method Validation

The following procedures were employed to investigate the analytical performance and efficacy of the methodology. Standard solutions of the analytes were made at the following concentrations: 0, 25, 50, 100, 250, and 500 ng/mL and their linearity was found to be 0.9994 for TCS and 0.9988 for TCC after four (*n* = 4) replicate analyses at all concentration levels. Spiked sample analysis was performed for concentrations of 0, 10, 25, 50, 100, and 250 pg/mg with linearity at 0.9910 for TCS and 0.9958 for TCC after five replicate analyses at all levels. Both the limit of detection (LOD) and limit of quantification (LOQ) were evaluated using the signal to noise ratio ≥ 3 for LOD and ≥10 for LOQ, respectively. Three replicate analyses of spiked samples (*n* = 3) were used for the evaluation of the recovery, accuracy and inter-day precision (%RSD) of the method at each spiked concentration level, 25, 50, 100 and 250 pg/mg for recovery and accuracy and 25, 100 and 250 pg/mg for inter-day precision. The method validation parameters are summarized in Table 2.

### 2.6. Statistical Analysis

GraphPad Prism version 9.2.0 (GraphPad Software, San Diego, CA, USA) was used to perform the statistical analysis. Due to the non-normal distribution of the data, as confirmed by the Shapiro–Wilk test, a nonparametric Kruskal–Wallis test with Dunn’s post hoc test was used to compare levels of compounds between specific regions. Differences were considered statistically significant at *p* < 0.05. In the statistical analysis, values below LOQ and LOD were taken into account as LOQ/2 and LOD/2, respectively.

## 3. Results

Considering all samples included in this study, regardless of the wild boars’ region of origin, TCS and TCC concentrations above the LOD were found in a relatively low percentage of samples (Table 3). Regarding TCC, levels above the LOD were detected in 14.81% of the studied samples (and above the LOQ in 12.96% of the samples). The minimum TCC concentration above the LOQ was 5.1 pg/mg, while the maximum reached 17.2 pg/mg. TCS concentrations above the LOD were observed in an even smaller proportion of samples. Specifically, such levels were detected in only two samples (3.70% of the samples included in the study). A TCS concentration exceeding the LOQ was found in just a single sample (1.85% of all samples), measuring 15.5 pg/mg. TCC levels above the LOD were found in four samples from males (13.79% of samples from males) and also in four samples from females (16.00% of samples from females). Both samples in which TCS levels were above the LOD were collected from females.

**Figure 3:**
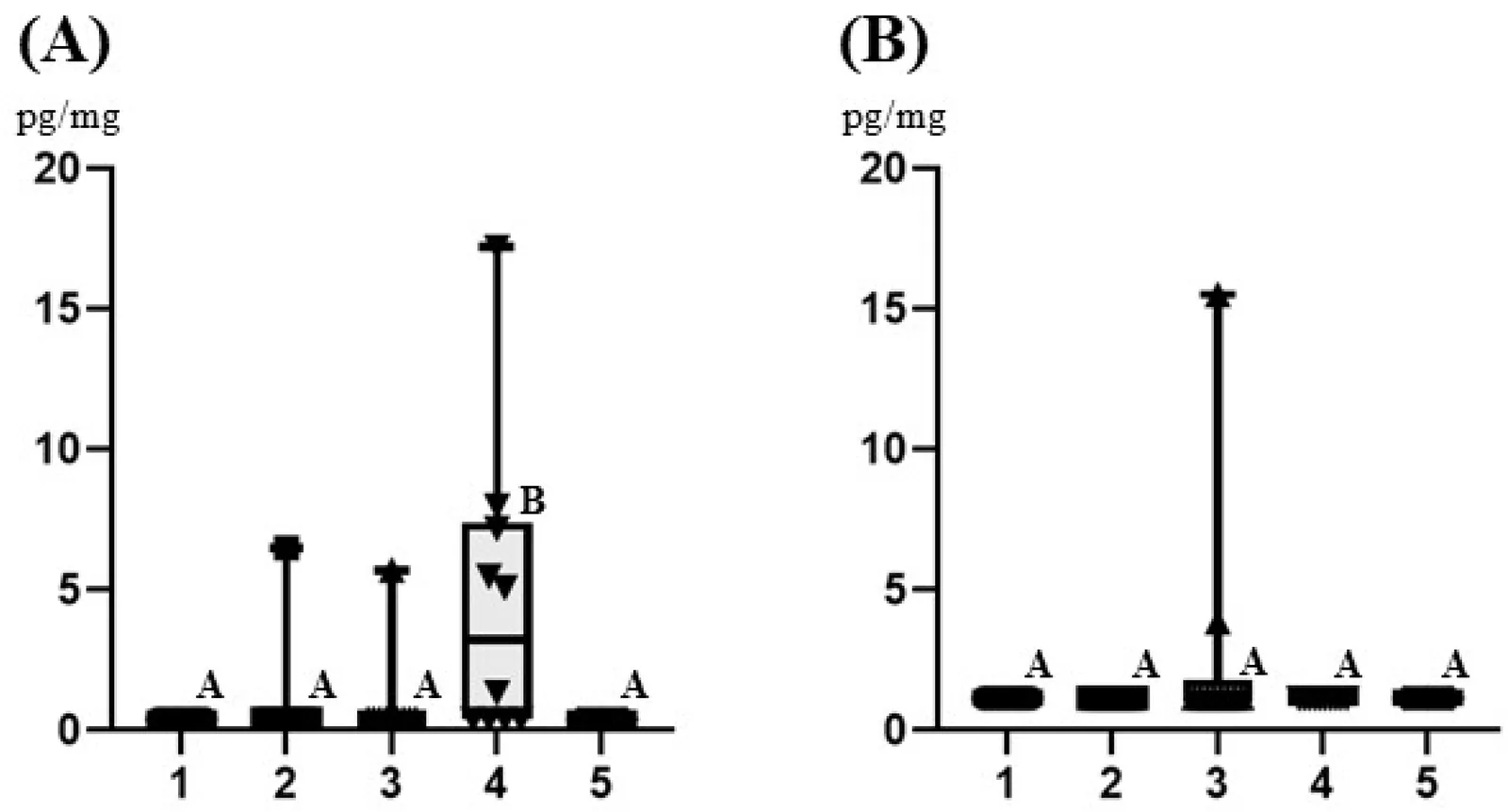
Concentration levels of (**A**) triclocarban (TCC) and (**B**) triclosan (TCS) in wild boar hair samples collected in various voivodeships of Poland: 1—West Pomeranian; 2—Pomeranian; 3—Kuyavian–Pomeranian; 4—Silesian; 5—Holy Cross. Statistically significant differences (*p* < 0.05) are marked with different letters, and values not showing such differences are marked with the same letter. Different shapes indicate substance levels in different voivodeships. Values below LOQ and LOD were taken into account as LOQ/2 and LOD/2, respectively. The figure was created using GraphPad Prism version 9.2.0 (GraphPad Software, San Diego, CA, USA).

The largest number of samples in which TCC was present at levels above the LOD was from the Silesian Voivodeship. TCC levels above the LOD were noted in 6 samples from this region (60% of all samples from this region). In one sample from this region, the TCC level was below the LOQ, and in the others, it ranged from 5.1 pg/mg to 17.2 pg/mg. The median level of TCC in samples from the Silesian Voivodeship was 3.23 pg/mg (mean 4.60 ± 5.37 pg/mg, 25th percentile below the LOD, 75th percentile 7.40 pg/mg). TCC concentrations exceeding the LOQ were also detected in one sample from the Kuyavian–Pomeranian Voivodeship and one from the Pomeranian Voivodeship. Conversely, median levels of TCC in both these regions were below the LOD, and the means were below the LOQ. No TCC concentrations above the LOD were found in any samples from the West Pomeranian or Holy Cross voivodeships. The TCC concentration levels in the Silesian Voivodeship were statistically significantly higher than in the other voivodeships (Figure 3A).

In turn, both samples in which TCS levels were higher than the LOD were collected from female wild boars in the Kuyavian–Pomeranian Voivodeship. The median level of TCS in this voivodeship was below the LOD, and the mean was below the LOQ. Differences in TCS concentrations between the Kuyavian–Pomeranian and other voivodeships were not statistically significant (Figure 3B).

However, it should be noted that in the present study, the levels of TCC and TCS in most samples were below the LOD and LOQ. For statistical analysis, these values were assumed to be LOD/2 or LOQ/2, respectively. This method of presenting values is widely accepted; however, it should be kept in mind that a high percentage of such values (as is the case in this study) can significantly affect the results of the statistical analysis.

## 4. Discussion

Based on previous studies, both TCC and TCS are recognized as ubiquitous environmental contaminants, occurring in various environmental compartments in many regions of the world [18,19,20]. The presence of these substances has also been documented in both human and animal organisms [35,36,37,38,40]. Research on the occurrence of TCS in the natural environment and living organisms in Poland is very limited. However, studies have shown the presence of TCS in surface waters [52,53] and in the bodies of people living in various regions of the country, both more and less urbanized [47,54,55]. As for TCC, no studies have yet been conducted on the occurrence of this substance in living organisms or the environment in Poland.

Nevertheless, the widespread and well-documented environmental occurrence of TCC and TCS suggests that wildlife is also significantly exposed to these compounds. Exposure to anthropogenic compounds primarily concerns wildlife inhabiting areas near human settlements. This is particularly true for wild boars, which are increasingly found even in large urban areas, and often forage at landfill sites [41,42]. Furthermore, previous studies have demonstrated that wild boars are exposed to other anthropogenic endocrine-disrupting compounds, including bisphenols, parabens, and phthalates [43,44,56]. However, the present study clearly demonstrated that only a relatively small proportion of the analyzed wild boar hair samples contained concentrations of the investigated compounds above the LOD. These findings may suggest that wild boars are exposed to TCC and TCS only to a limited extent. This contrasts markedly with the situation observed for the aforementioned bisphenol A, some parabens, and phthalates, which are commonly detected in wild boar hair [43,44,56].

To date, TCC and TCS levels in hair have only been determined in humans, but the number of these studies, especially in the case of TCC, is highly limited (Table 4). Comparing the results obtained in the present study with those found in humans, it can be stated that both the levels of TCC and TCS in hair and their detection frequency above the LOD are lower in wild boars. This situation may be due to the fact that these substances are anthropogenic contaminants, and humans are more exposed to them through their lifestyle, use of cosmetics, and other consumer products that may contain them.

Although there are relatively numerous studies on TCC and TCS levels in humans, as well as in domestic and wild animals (Supplementary Materials—Table S1), they have been conducted on entirely different matrices, such as nails [31,60], urine [31,61,62,63,64], muscle [40,65,66,67], seminal plasma [68] and serum samples [69,70,71]. Given that hair as a matrix possesses vastly different properties, a direct and logical comparison of the results obtained in the present study with previously observed levels in animals is virtually impossible. This is further supported by literature demonstrating significant discrepancies between TCC and TCS levels in hair compared to other matrices [31].

During the present study, it was demonstrated that TCC was mainly present in animal hair samples originating from the Silesian Voivodeship. It should be emphasized that this is a highly industrialized and urbanized area, featuring numerous large urban centers that form extensive agglomerations. The Silesian Voivodeship is also characterized by the highest population and the highest population density among the studied regions (Table 1). Furthermore, previous studies have shown that the natural environment in the Silesian Voivodeship is contaminated with various substances of anthropogenic origin and harmful elements [72,73,74]. Although TCC and TCS levels in the environment of the Silesian Voivodeship have not been monitored so far, the obtained results confirming the presence of TCC in wild boar hair samples from this region strongly suggest environmental contamination with this compound. The high degree of urbanization is likely the cause of the TCC presence in the hair of wild boars from this region, especially since previous studies suggest a positive correlation between the degree of urbanization and environmental contamination with this compound [75,76]. Regarding the remaining voivodeships covered by this study, TCC concentrations above the LOD were observed in individual specimens, namely in one animal from the Kuyavian–Pomeranian Voivodeship and one from the Pomeranian Voivodeship. These voivodeships, similar to the other voivodeships covered by the study, exhibit a significantly lower degree of urbanization and industrialization than the Silesian Voivodeship (Table 1). Meanwhile, the presence of TCC above the LOD in individual samples may suggest that local environmental contamination with this substance is high. Such local sources of contamination are difficult to identify without comprehensive environmental studies and may, for instance, be associated with illegal waste dumping sites in forests where the animals live or point-source pollution of small water bodies, such as ponds [77].

In the present study, TCS concentrations above the LOD were observed in wild boar hair even less frequently than TCC concentrations. Such concentrations were detected in only two samples originating from the Kuyavian–Pomeranian Voivodeship, which exhibits a relatively low degree of urbanization and industrialization. This fact may suggest that the higher exposure of wild boars to TCS is sporadic and is likely related to local factors, such as those mentioned above [78].

The results obtained in the present study are intriguing for two reasons. Firstly, previous studies have demonstrated that the natural environment is frequently co-contaminated with both TCC and TCS, and living organisms are simultaneously and equally exposed to both substances [31,79,80]. However, the present study did not confirm this fact, as not a single analyzed sample showed the simultaneous presence of TCC and TCS above the LOD values.

Secondly, it is interesting to note that no hair sample from animals in the Silesian Voivodeship—which, as previously mentioned, has the highest degree of urbanization and industrialization among all studied voivodeships—contained TCS concentrations above the LOD. Explaining these issues is impossible without comprehensive toxicological and environmental studies that clarify all aspects of environmental contamination with both compounds investigated in this study, and that identify the primary local sources of these compounds in the environment across individual voivodeships. An important aspect of these future studies will also be to understand the exact metabolism of TCC and TCS in various wild animal species, including wild boars, as metabolic processes could have also influenced the obtained results. Specifically, TCS is known to undergo rapid biotransformation in mammalian systems via phase I hydroxylation and efficient phase II conjugation (primarily sulfation and glucuronidation), leading to its faster clearance [81]. In contrast, TCC is a highly lipophilic carbanilide derivative with a more stable molecular backbone that resists initial degradation, making it more prone to accumulation in lipid-rich and keratinous structures such as hair [82]. Therefore, the observed differences in the detection frequencies of TCC and TCS above the LOD may largely depend on the distinct metabolic pathways and tissue distribution dynamics of these two compounds.

Although TCC and TCS concentrations above the LOD were observed in only a relatively small percentage of the studied samples, the presence of these substances indicates that wild boars in certain limited areas are continuously exposed to these compounds. It should be emphasized that hair is a specific matrix in bioaccumulation monitoring. Substances accumulate inside the hair, binding with keratin, and their levels do not undergo short-term changes [48]. Therefore, hair analysis allows for the assessment of long-term exposure to a given substance. It is estimated that the so-called window of exposure, which can be examined by analyzing a hair sample, can range from a few weeks to even several years, depending on the species and the length of the hair, as well as the hair growth rate [83,84]. It is worth noting that the term “half-life” is not applicable to studies investigating substance levels in hair (including TCC and TCS). This is because, unlike serum or urine samples, hair is a metabolically inactive matrix. Therefore, once TCS and TCC are incorporated into the hair shaft during its formation, they are locked within the keratinized matrix. Because of this lack of metabolic activity, these compounds are protected from metabolism and elimination. Consequently, hair serves as a matrix reflecting long-term, chronic exposure. Furthermore, TCS and TCC exhibit a high affinity for lipids and proteins, suggesting the high chemical stability of these substances within the hair matrix.

In contrast to hair, the analysis of blood or urine samples allows for the investigation of short-term changes in exposure, as the detection window for these matrices ranges from a few hours to a few days [85]. In the present study, undercoat hair samples were analyzed. The undercoat appears around mid-August and grows until mid- or late September. After this period, hair growth is arrested, which also inhibits the accumulation of substances within the hair shaft [86]. Taking this into account, this experiment investigated an approximately 1- to 1.5-month exposure of the animals to TCC and TCS.

Hair as a biomonitoring matrix also has certain limitations. The most significant of these is the fact that substances can incorporate into the hair structure via two distinct pathways [48]. The first is the endogenous (internal) pathway, in which substances absorbed by the body reach the hair follicle through blood vessels and enter the hair shaft. The second is the exogenous (external) pathway, which involves the direct penetration of substances into the hair from the external environment. Unfortunately, when analyzing substance concentrations inside the hair, it is impossible to determine what fraction entered through the endogenous pathway versus the exogenous one.

In addition to the limitations arising from the characteristics of hair as a matrix, the present study has other limitations.

One of these is the relatively small sample number, meaning the lack of additional samples from other areas as well as the lack of samples collected in every season. An analysis of such samples would certainly enrich this research. This is due to the limited number of legal hunts in the studied regions and the limited willingness of local hunting clubs to cooperate. The second limitation is the lack of animal classification into age groups, as accurately determining the age of wild boars (especially older individuals) is relatively difficult and prone to significant error. Another significant limitation is the lack of wild boar migration monitoring in Poland, which makes it impossible to trace the exact origin of the animals. On the other hand, wild boars are sedentary animals that display strong site fidelity to their home range (known as a refuge) and may inhabit a single area for many years, provided they have stable access to food and shelter [87]. Therefore, it can be assumed that the location where a given animal was hunted corresponds to the area where it had lived for an extended period. A certain limitation of the study is also the lack of comparison between samples from different periods of the year. However, the peak of wild boar hunting in Poland occurs in autumn, and samples from this period are the easiest to obtain. Despite these limitations, this study, as the first analysis of TCS and TCC levels in wild boar hair, enriches our knowledge regarding the exposure of wild terrestrial mammals to these substances.

## 5. Conclusions

Despite the widespread presence of TCC and TCS in the natural environment, the present study revealed the presence of these compounds above the LOD in only a relatively small percentage of wild boar hair samples. These results may indicate that wild boars are not exposed to high levels of these compounds. TCC at concentrations above the LOD was found more frequently in the studied samples than TCS, particularly in animals living in highly urbanized and industrialized areas. The obtained results also suggest that local, difficult-to-identify factors may be responsible for the exposure of animals to TCC and TCS, especially in less urbanized areas. However, clarifying all aspects related to the exposure of wild terrestrial mammals to TCC and TCS requires further comprehensive research.

## Figures and Tables

**Table 1:** Regions included in the study.

No.	1	2	3	4	5
Voivodeship name	West Pomeranian	Pomeranian	Kuyavian–Pomeranian	Silesian	Holy Cross
Area (in 1000 km^2^)	22.8	18.3	17.9	12.3	11.7
% of urbanized area	55.2	65	61.1	77.6	44.8
Human population density (persons/km^2^)	73	128	114	361	104
% of agricultural land	38.3	47.1	61.1	29.8	48.2
Industrialization ^(1)^	Medium industrialized	Highly industrialized	Medium industrialized	Highly industrialized (mining)	Low industrialized
Annual average PM10 (a) and PM2.5 (b) concentrations (μg/m^3^)	(a) 10–19 (b) 10–13	(a) 14–19 (b) 10–13	(a) 22–27 (b) 15–18	(a) 26–36 (b) 20–23	(a) 23–29 (b) 20–21

Data from 2021 according to Central Statistical Office in Poland, https://stat.gov.pl/ (accessed on 10 May 2026). ^(1)^ Degree of industrialization according to scale developed by Bal-Domańska and Stańczyk [50].

**Table 2:** Validation parameters of the applied methodology.

	TCS	TCC	No. of Repetitions
Mean % recovery	106.5	85.2	*n* = 3
Mean % accuracy	111.1	100.0	*n* = 3
Precision (%RSD)	13.4	14.3	*n* = 3
LOD (pg/mg)	2.3	0.8	*n* = 2
LOQ (pg/mg)	7.7	2.7	*n* = 2
r^2^ (standard curves)	0.9994	0.9988	*n* = 4
r^2^ (spiked curves)	0.9910	0.9958	*n* = 5

TCS—triclosan; TCC—triclocarban; LOD—limit of detection; LOQ—limit of quantification.

**Table 3:** Concentration levels (pg/mg) of triclocarban (TCC) and triclosan (TCS) in individual samples included in the study and cumulative data (LOD—limit of detection; LOQ—limit of quantification).

Sample No. (Sampling Year)	Animal Gender	Region	TCC	TCS
1 (2021)	F	West Pomeranian	<LOD	<LOD
2 (2021)	F		<LOD	<LOD
3 (2021)	M		<LOD	<LOD
4 (2021)	M		<LOD	<LOD
5 (2022)	M		<LOD	<LOD
6 (2022)	M		<LOD	<LOD
7 (2022)	F		<LOD	<LOD
8 (2021)	M		<LOD	<LOD
9 (2021)	M		<LOD	<LOD
10 (2022)	F		<LOD	<LOD
11 (2021)	F	Pomeranian	<LOD	<LOD
12 (2021)	M		<LOD	<LOD
13 (2021)	F		<LOD	<LOD
14 (2022)	F		<LOD	<LOD
15 (2022)	M		<LOD	<LOD
16 (2022)	F		<LOD	<LOD
17 (2022)	F		<LOD	<LOD
18 (2022)	M		<LOD	<LOD
19 (2022)	F		<LOD	<LOD
20 (2022)	F		6.5	<LOD
21 (2021)	M	Kuyavian–Pomeranian	<LOD	<LOD
22 (2021)	M		<LOD	<LOD
23 (2021)	F		<LOD	<LOD
24 (2021)	M		5.7	<LOD
25 (2021)	M		<LOD	<LOD
26 (2021)	F		<LOD	<LOD
27 (2022)	F		<LOD	15.5
28 (2022)	M		<LOD	<LOD
29 (2022)	M		<LOD	<LOD
30 (2022)	F		<LOD	<LOQ
31 (2021)	M	Silesian	<LOD	<LOD
32 (2021)	M		5.5	<LOD
33 (2021)	M		8.0	<LOD
34 (2021)	M		<LOQ	<LOD
35 (2021)	F		7.2	<LOD
36 (2022)	F		5.1	<LOD
37 (2022)	M		<LOD	<LOD
38 (2022)	M		<LOD	<LOD
39 (2022)	F		17.2	<LOD
40 (2022)	F		<LOD	<LOD
41 (2021)	F	Holy Cross	<LOD	<LOD
42 (2021)	F		<LOD	<LOD
43 (2021)	M		<LOD	<LOD
44 (2021)	M		<LOD	<LOD
45 (2022)	M		<LOD	<LOD
46 (2022)	M		<LOD	<LOD
47 (2022)	M		<LOD	<LOD
48 (2022)	F		<LOD	<LOD
49 (2022)	F		<LOD	<LOD
50	M		<LOD	<LOD
51	F		<LOD	<LOD
52	M		<LOD	<LOD
53	F		<LOD	<LOD
54	M		<LOD	<LOD
Cumulative data				
Minimum			<LOD	<LOD
25% percentile			<LOD	<LOD
median			<LOD	<LOD
75% percentile			<LOD	<LOD
Maximum			17.2	15.5
Mean			<LOQ	<LOD

**Table 4:** Concentration levels (pg/mg) and % of samples with the concentration above the LOD in human hair according to previous studies.

Country	n	% Samples > LOD	Range	Median	Mean (±SD)	Reference
TRICLOSAN (TCS)						
China	60	100	6.48–5140	110	507 ± 1040	[31]
Germany	4	25	1840–1870	*	*	[57]
Greece	100	Adults				[49]
		92	13.2–8564.9	181.6	687.0 ± 1501	
		Children				
		90.3	3.6–7656.6	48.8	275.2 ± 1005.7	
	95	98.9	8.8–8070.2	61.6	245.0 ± 859.5	[58]
	49	I trimester of pregnancy				[59]
		93.9	34–356	71	208 ± 241	
		II trimester of pregnancy				
		93.9	51–540	116	310 ± 412	
		III trimester of pregnancy				
		93.9	38–447	113	275 ± 345	
Poland	30	96.7	37.9–3386.5	103	402.6 ± 803.6	[47]
TRICLOCARBAN (TCC)						
China	60	100	1.88–3280	14.0	72.2 ± 421	[31]

* The authors do not provide the mean and median values. *n*—number of analyzed samples.

## Data Availability

All data are available in the manuscript.

## Supplementary Materials

The following supporting information can be downloaded at: https://www.mdpi.com/article/10.3390/ani16182863/s1, Table S1. Concentration levels (range or median) in pg/mg for solid matrices, and ng/mL for liquid matrices of triclosan (TCS) and triclocarban (TCC) in humans, domestic, and wild animals according to selected previous studies.
