# Supplementary material for: Are Wild Boars Exposed to Triclosan and Triclocarban?
Source: Animals (Basel). 2026 Sep 11;16(18):2863. doi: 10.3390/ani16182863 (PMC13603304; doi:10.3390/ani16182863)
Supplement: Supplementary file 1 [file animals-16-02863-s001.zip › animals-4472861-supplementary.pdf]

Supplementarny materials for

Are wild boars exposed to triclosan and triclocarban?

Sławomir Gonkowski [1], Manolis Tzatzarakis [2], Elena Vakonaki [2] and Liliana Rytel [3]

[1]Department of Clinical Physiology, Faculty of Veterinary Medicine, University of Warmia and Mazury in Olsztyn, Olsztyn, Poland.
[2]Laboratory of Toxicology, School of Medicine, University of Crete, Heraklion, Crete, Greece.
[3]Department and Clinic of Internal Diseases, Faculty of Veterinary Medicine, University of Warmia and Mazury in Olsztyn, Olsztyn, Poland.

Table S1. Concentration levels (range or median) in pg/mg for solid matrices, and ng/mL for liquid matrices of triclosan (TCS) and triclocarban (TCC) in humans, domestic, and wild animals according to se-lected previous studies

Country	Matrix	TCS	TCC	Reference
**Humans**				
China	nails	6.6 -1488	9.5-7686.7	[1]
	nails	<LOD-8470	1.04-4080	[2]
	urine	<LOD-264	0.11-51.4	[2]
	serum	1,08	<LOD	[3]
Greece	urine	5.8	0.5	[4]
Puerto Rico	urine	8.4	0.1	[5]
USA	seminal plasma	10.6	<LOQ	[6]
	urine	7.84	0,02	[7]
**Dogs**				
USA	urine	<LOQ-3.9	ND	[8]
**Cats**				
USA	urine	<LOQ-2.2	<LOQ-0.3	[8]
**Deer mice**				
USA	muscles	ND	ND	[9]
	liver	ND-10.7	ND-33.3	
**Bald eagles**				
USA	plasma	0.82-1.8	0.27-0.58	[10]
**Birds (various species)**				
USA	eggs	4.1-37.9	3.3-31.4	[9]
**Fish (various species)**				
India	muscles	-	0.8-692	[11]
	muscles	ND - 589	ND-285.5	[12]
	plasma	<LOD-1.9	<LOD- 6.8	[13]
Indonesia	plasma	<LOD-11	<LOD-0,26	[13]
Philippines	muscles	0.021–507	0.004–157	[14]
Vietnam	plasma	<LOD-10	<LOD-3.3	[13]
**Earthworms**				
USA	body	5.9-42.8	ND-5.1	[9]

LOD: limit of detection; LOQ: limit of quantification; ND: not detected

1. Shi, Y.; Liu, X.; Zhang, J.; Shao, B. Analysis of triclosan and triclocarban in human nails using isotopic dilution liquid chromatography-tandem mass spectrometry. *J. Chromatogr. B Analyt. Technol. Biomed. Life. Sci*. **2013**, *934*, 97-101. doi: 10.1016/j.jchromb.2013.07.003.

2. Tian, X.; Huang, K.; Liu, Y.; Jiang, K.; Liu, R.; Cui, J.; Wang, F.; Yu, Y.; Zhang, H.; Lin, M.; Ma, S. Distribution of phthalate metabolites, benzophenone-type ultraviolet filters, parabens, triclosan and triclocarban in paired human hair, nail and urine samples. *Environ. Pollut*. **2023**, *333*,122083. doi: 10.1016/j.envpol.2023.122083.
3. Li, A.; Zhuang, T.; Zhu, Q.; Song, M.; Liao, C.; Jiang, G. Concentration and distribution of parabens, triclosan, and triclocarban in pregnant woman serum in China. *Sci. Total. Environ*. **2020**, *710*, 136390. doi: 10.1016/j.scitotenv.2019.136390.
4. Asimakopoulos, A.G.; Thomaidis, N.S.; Kannan, K. Widespread occurrence of bisphenol A diglycidyl ethers, p-hydroxybenzoic acid esters (parabens), benzophenone type-UV filters, triclosan, and triclocarban in human urine from Athens, Greece. *Sci. Total. Environ.* **2014**, *470-471*, 1243-9. doi: 10.1016/j.scitotenv.2013.10.089.
5. Ashrap, P.; Watkins, D.J.; Calafat, A.M.; Ye, X.; Rosario, Z.; Brown, P.; Vélez-Vega, C.M.; Alshawabkeh, A.; Cordero, J.F.; Meeker, J.D. Elevated concentrations of urinary triclocarban, phenol and paraben among pregnant women in Northern Puerto Rico: Predictors and trends. *Environ. Int.* **2018,** *121(Pt 1),* 990-1002. doi: 10.1016/j.envint.2018.08.020.
6. Smarr, M.M.; Kannan, K.; Sun, L.; Honda, M.; Wang, W.; Karthikraj, R.; Chen, Z.; Weck, J.; Buck Louis, G.M. Preconception seminal plasma concentrations of endocrine disrupting chemicals in relation to semen quality parameters among male partners planning for pregnancy. *Environ. Res*. **2018**, *167*, 78-86. doi: 10.1016/j.envres.2018.07.004.
7. Iyer, A.P.; Xue, J.; Honda, M.; Robinson, M.; Kumosani, T.A.; Abulnaja, K.; Kannan, K. Urinary levels of triclosan and triclocarban in several Asian countries, Greece and the USA: Association with oxidative stress. *Environ. Res*. **2018**, *160*, 91-96. doi: 10.1016/j.envres.2017.09.021.
8. Karthikraj, R.; Lee, S.; Kannan, K. Biomonitoring of exposure to bisphenols, benzophenones, triclosan, and triclocarban in pet dogs and cats. *Environ. Res*. **2020**, *180*, 108821. doi: 10.1016/j.envres.2019.108821.
9. Sherburne, J.J.; Anaya, A.M.; Fernie, K.J.; Forbey. J.S.; Furlong, E.T.; Kolpin, D.W.; Dufty, A.M.; Kinney, C.A. Occurrence of Triclocarban and Triclosan in an Agro-ecosystem Following Application of Biosolids. *Environ. Sci. Technol*. **2016**, *50*, 13206-13214. doi: 10.1021/acs.est.6b01834.
10. Elliott, S.M.; Route, W.T.; DeCicco, L.A.; VanderMeulen, D.D.; Corsi, S.R.; Blackwell, B.R. Contaminants in bald eagles of the upper Midwestern U.S.: A framework for prioritizing future research based on in-vitro bioassays. *Environ. Pollut.* **2019**, *244,* 861-870. doi: 10.1016/j.envpol.2018.10.093.
11. Vimalkumar, K.; Arun, E.; Krishna-Kumar, S.; Poopal, R.K.; Nikhil, N.P.; Subramanian, A.; Babu-Rajendran, R. Occurrence of triclocarban and benzotriazole ultraviolet stabilizers in water, sediment, and fish from Indian rivers. *Sci. Total. Environ*. **2018**, *625*, 1351-1360. doi: 10.1016/j.scitotenv.2018.01.042.
12. Das Sarkar, S.; Nag, S.K.; Kumari, K.; Saha, K.; Bandyopadhyay, S.; Aftabuddin, M.; Das, B.K. Occurrence and Safety Evaluation of Antimicrobial Compounds Triclosan and Triclocarban in Water and Fishes of the Multitrophic Niche of River Torsa, India. *Arch. Environ. Contam. Toxicol*. **2020**, *79*, 488-499. doi: 10.1007/s00244-020-00785-0.
13. Nozaki, K,; Tanoue, R.; Kunisue, T.; Tue, N.M.; Fujii, S.; Sudo, N.; Isobe, T.; Nakayama, K,; Sudaryanto, A.; Subramanian, A.; Bulbule, K.A.; Parthasarathy, P.; Tuyen, L.H.; Viet, P.H.; Kondo, M.; Tanabe, S.; Nomiyama, K. Pharmaceuticals and

personal care products (PPCPs) in surface water and fish from three Asian countries: Species-specific bioaccumulation and potential ecological risks. *Sci. Total. Environ.* **2023,** *866,* 161258. doi: 10.1016/j.scitotenv.2022.161258.

14. Ramaswamy, B.R.; Kim, J.W.; Isobe, T.; Chang, K.H.; Amano, A.; Miller, T.W.; Siringan, F.P.; Tanabe, S. Determination of preservative and antimicrobial compounds in fish from Manila Bay, Philippines using ultra high performance liquid chromatography tandem mass spectrometry, and assessment of human dietary exposure. *J. Hazard. Mater*. **2011**, *192*, 1739-45. doi: 10.1016/j.jhazmat.2011.07.006.
